# [Retracted] Serum miR‑338‑5p has potential for use as a tumor marker for retinoblastoma

**DOI:** 10.3892/ol.2024.14347

**Published:** 2024-03-19

**Authors:** Peng Zhou, Xuemin Li

Oncol Lett 18: 307–313, 2019; DOI: 10.3892/ol.2019.10331

Following the publication of this paper, it was drawn to the Editor's attention by a concerned reader that the Transwell cell migration and invasion assay data shown in Fig. 4A and B on p. 312 had already appeared in several previously published articles written by different authors at different research institutes. In addition, the second (more senior) author listed on the paper (Xuemin Li), who was also included as the author for correspondence, claimed that he had no knowledge of this paper's submission, and requested that it be withdrawn from the publication.

Owing to the fact that the contentious data in the above article had already been published prior to its submission to *Oncology Letters*, the Editor has agreed with the request that this paper should be retracted from the Journal. The Editor apologizes to the readership for any inconvenience caused.

